# Geospatial analysis of type 2 diabetes mellitus and hypertension in South Sulawesi, Indonesia

**DOI:** 10.1038/s41598-023-27902-y

**Published:** 2023-01-16

**Authors:** Andi Alfian Zainuddin, Amran Rahim, Sri Ramadany, Himawan Dharmayani, Hedi Kuswanto, Rais Reskiawan A. Kadir, Andi Afdal Abdullah, Haerani Rasyid

**Affiliations:** 1 Faculty of Medicine, Hasanuddin University, Makassar, 90245 Indonesia; 2 Faculty of Mathematics and Natural Sciences, Hasanuddin University, Makassar, 90245 Indonesia; 3 Social Insurance Administration Organization, Jakarta, 10510 Indonesia

**Keywords:** Epidemiology, Population screening, Health care

## Abstract

The spatial variation of type 2 diabetes mellitus (T2DM) and hypertension and their potential linkage were explored in South Sulawesi Province, Indonesia. The Global Moran’s I and regression analysis were utilized to identify the characteristics involved. The methods were performed based on T2DM and hypertension data from 2017 and 2018 acquired from Social Health Insurance Administration in Indonesia. The spatial variation of T2DM and hypertension showed that the prevalence rate of T2DM and hypertension tends to occur randomly (*p* = 0.678, *p* = 0.711, respectively). By utilizing Generalized Poisson Regression Analysis, our study showed a significant relationship between T2DM and hypertension (*p* ≤ 0.001). This research could help policy makers to plan and support projects with the aim of overcoming the risk of T2DM and hypertension.

## Introduction

Non-communicable diseases (NCD) are one of the health problems of national and global concern currently. Data from the World Health Organization (WHO) showed that out of the 57 million deaths that year, 41 million or almost two-thirds of the total were caused by NCD^1^. In countries with low to middle economic levels, 29% of deaths in people less than 60 years old were caused by NCD^1^. Type 2 diabetes mellitus (T2DM) is one of four NCD priorities because of several detrimental complications, such as blindness, heart attack, stroke, kidney disorder and leg amputation^1^. The prevalence of T2DM worldwide reached 415 million people in 2015 and it is estimated that by 2040 the number of people with T2DM will account to 642 million. Blood sugar levels greater than optimal value (normal fasting blood glucose concentration are between 70 mg/dL (3.9 mmol/L) and 100 mg/dL (5.6 mmol/L) resulted in an additional 2.2 million deaths, mainly through the increased risk for cardiovascular and other linked diseases^1^.

In the last few decades, Indonesia has faced the problem of a triple-burden as infectious diseases and re-emerging of other diseases remain, while new diseases continue to emerge frequently. Importantly, the prevalence of NCD has also increased with the rising prevalence of T2DM recently from 6.9 to 8.5% and hypertension from 25.8 to 34.1%^2^. There may be a link between two diseases, as diabetic patients can experience an increase in blood pressure and 40–60% of diabetic cases often show high blood pressure. Furthermore, T2DM and hypertension can cause a variety of complications without symptoms^3^. The interaction between hypertension and T2DM can lead to the development of stroke and myocardial infarction^3^.

One of the important controls and prevention strategies for T2DM and hypertension would be to apply spatial analysis to find areas with a high risk of both diseases and make early prevention efforts. Spatial analysis provides more information about risk based on spatial variation, making prediction more accurate. Knowledge about the risk of T2DM and hypertension in each district or city area can help health agencies carry out activities and programs to effectively prevent these two diseases^4^.

The current study aimed to determine the level of risk of T2DM and hypertension in each district or city area in South-Sulawesi Province, which can be performed by identifying spatial cluster in the number of people with the disease, carrying out geospatial analysis and applying a Generalized Poisson Regression (GPR) model to determine the potential effect of T2DM on hypertension.

## Materials and methods

### Ethics approval

This study was reviewed and approved by Ethics Committee of Medical Research of Faculty of Medicine Hasanuddin University (556/UN4.6.4.5.31/PP36/2021). This study was conducted in accordance with the Helsinki declaration. Informed consent was waived by the Ethics Committee of Medical Research of Faculty of Medicine Hasanuddin University. The dataset in the current study was acquired from national Social Health Insurance Administration Body (BPJS Kesehatan).

### Study area

**Figure 1:**
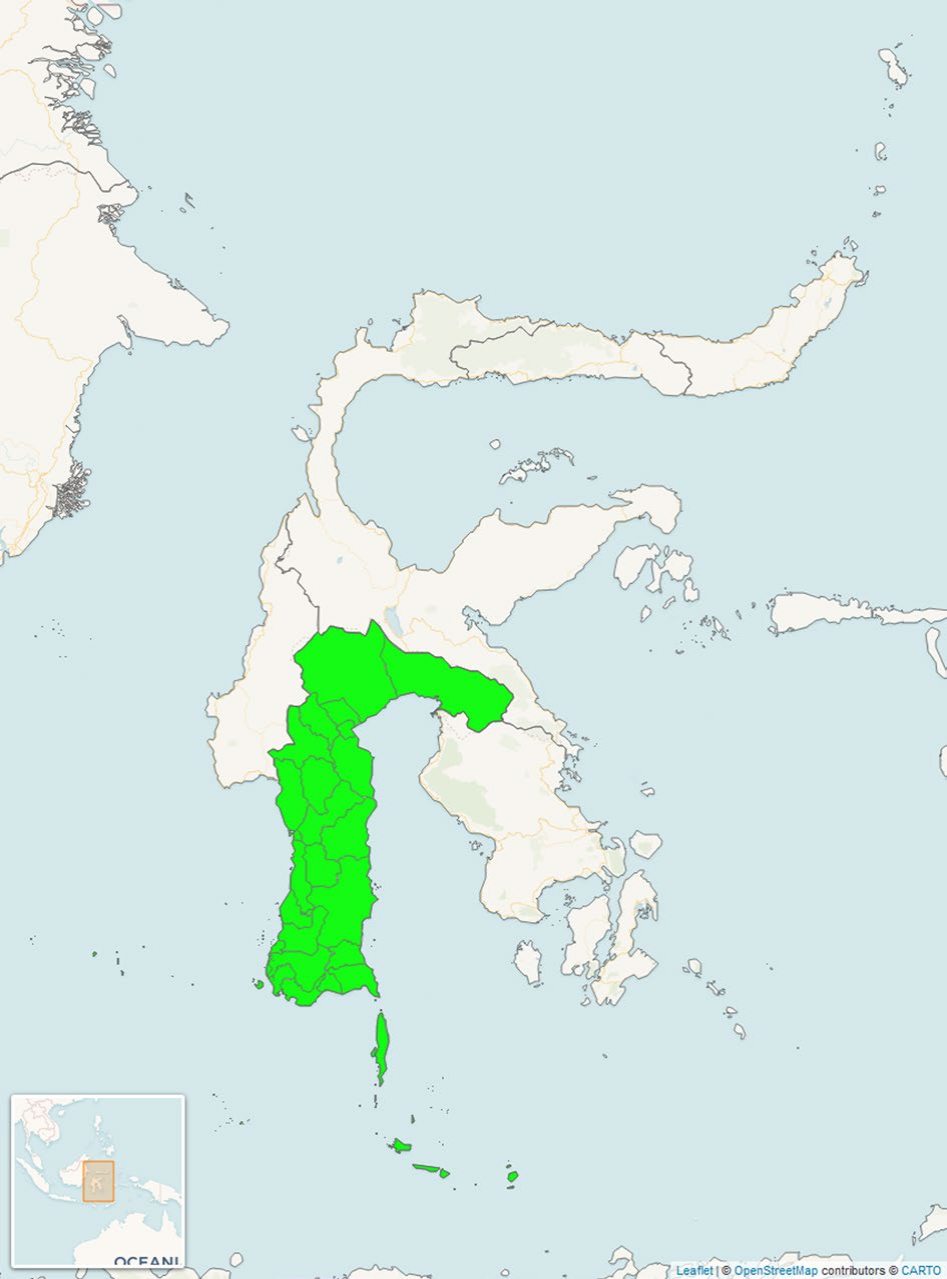
The map of the research area. The green area indicated the South Sulawesi Province where the study was conducted. This figure was generated by using R Basic version: 4.2.2 (https://cran.r-project.org/bin/windows/base/) and R Studio Desktop version: 2022.07.2 + 576 (https://posit.co/download/rstudio-desktop/) for windows operating system. We also utilized several packages to produce this figure such as sf package (https://r-spatial.github.io/sf/), dplyr package (https://github.com/tidyverse/dplyr), rgdal package (http://rgdal.r-forge.r-project.org/), tmap package (https://github.com/r-tmap/tmap) and leaflet package (https://rstudio.github.io/leaflet/).

South Sulawesi Province is one of the provinces in Indonesia with a high population number. The total population in 2020 was around 9 million people^5^. The total area covers about 46.717 km^2^. Figure 1 shows the location of the research area which coincides with South Sulawesi Province.

### Data utilized

The data used in this study refer to health insurance participants who suffered from T2DM and hypertension for the period of January 2017 to December 2018 consisted of 496 people with T2DM and 2597 people with hypertension, which spread all over 24 districts and cities in the South Sulawesi Province. In this research the districts and the cities denote a spatial unit of analysis. The data was generated from Social Health Insurance Administration Body, a government organization established to provide health insurance program for Indonesian. The population data were obtained from the Central Bureau of Statistics of South Sulawesi Province, locally known as Badan Pusat Statistik (BPS) which is a non-departmental government institute in Indonesia responsible for conducting statistical surveys. The daily data of the health insurance participants were accumulated by month and the prevalence rate was calculated per 100,000 people.

The daily data for T2DM and hypertension used in this study were processed based on the prevalence rate data for the occurrences of the two diseases. The prevalence rate was calculated for every 100,000 population by the formula:

1 $${\text{Prevalence}}\,{\text{rate}}\,\left( {\text{i}} \right) = \frac{The\,\,number\,\,of\,\, infected\,\, at\,\,district - i}{{The\,\,number\,\,of\,\,Population\,\,at\,\,district - i}}\, \times \,{100,000}$$

The prevalence rate was calculated annually and the prevalence rate for the 2017–2018 period is the average value for both 2017 and 2018 time periods in each district/city. In addition, we also calculated the minimum, maximum, mean and standard deviation of the prevalence rate in both diseases.

#### Statistical analysis

In this paper, we utilized Spatial Regression (SR) and Generalized Poisson Regression (GPR) models to examine the relationship between hypertension and T2DM. The models were fitted using the maximum likelihood method to assess the associations of the number of hypertension cases as the dependent variable and the number of TD2M cases as the independent variable. The inference procedure from the GPR models is performed using the coefficients of the T2DM variable and their standard errors. If the interval of estimated coefficients (estimated coefficients value − standard error; estimated coefficients value + standard error) contains zero value, then the independent variable insignificantly affect the dependent variable. Otherwise, the independent variable has a significant effect on the dependent variable.

The inference procedure was also conducted by calculating the probability value (*p-*value). If the *p-*value is less than the significance level (0.05) then the independent variable has a significant influence on the dependent variable. Otherwise, independent variables have a significant influence on a dependent variable. All the statistical analyses were performed using a 5% significance level and R-Studio version 1.2.5033 as a computation tool.

### Natural break classification method

The natural break method is a measurable procedure to identify cluster of values that are characteristic in data distribution. The natural break method using an algorithm that reduces the variance within classes and maximizes variances between classes. The natural break algorithm results may be expressed in a map of colors in gradation.

### Spatial cluster analysis

Generally, to identify spatial clusters of disease, Global Moran’s I statistics were used. Global Moran’s I is a global spatial autocorrelation statistic that identified correlation between one variable at location and different variable at the neighboring locations. If Global Morans I statistics was significant, we used Local Morans *I* and Getis-Ord Gi*^6,7^ to determine areas characterized as hotspots (concentrations) and coldspots (absence) with regard to T2DM and hypertension. This classifies the spatial patterns into clusters and outliers where the former can either be positive (hotspots) or negative (coldspots); while the latter are spatial objects whose attribute values are distinctly different from those of their spatial neighbors.

### Regressions models

We measured the effect of T2DM on hypertension using the SR^6^ and the GPR models^8^. In this study, we assessed the relationship between the number of T2DM and hypertension cases using the SR analysis as well as the GPR model, where T2DM is set as independent variables and hypertension is the dependent variable. The hypothetical tests are made to make a valid conclusion as to whether independent variables affect that dependent variable or not. The SR model is used to see if there is spatial contribution to hypertension. The GPR used to the dependent variable as count. It matched the number of hypertensive cases in the form of count. The spatial regression model is a regression model with spatial dependence through response variables (Spatial Lag Model/SLM) or in the components of random error (Spatial Error Model/SEM). In contrast. the classic regression model (Eq. 1) has no spatial dependency. The spatial lag model is stated in Eq. (2) and the spatial error model is stated in Eq. (3) as follows:

2 $$Y =\uprho {\mathbf{W}}Y + {\mathbf{X}}\beta + \varepsilon$$

3 $$Y = {\mathbf{X}}\beta + \, ({\mathbf{I}} - {\mathbf{W}}\uplambda )^{ - 1} u$$

where *u* = **W***u* + *ε*; *Y* the response variable; **X** the predictor variable; **W** the matrices of normalized weight spatial; *β* the coefficient of the predictor variable; *ε* a random error component; **I** the identity matrices; *u* a spatial random error; ρ the spatial effect of SLM; and λ the spatial effect of SEM. If ρ or λ is not significantly different from zero (*p-*value > 0.05) then there is no spatial dependency.

### The generalized Poisson regression (GPR) model

Let *Y*_*i*_ be a count response variable that followed the GPR distribution. The probability function of *Y*_*i*_ denoted as

$$f(y_{i} ,\mu_{i} ,a) \, = \left( {\frac{{\mu_{i} }}{{1 + \alpha \mu_{i} }}} \right)\frac{{\left( {1 + \alpha y_{i} } \right)^{{y_{i} - 1}} }}{{y_{i} !}}exp\left[ {\frac{{ - \mu_{i} \left( {1 + \alpha y_{i} } \right)}}{{\left( {1 + \alpha \mu_{i} } \right)}}} \right]$$

where *y*_*i*_ = 0, 1, 2, …; and *µ*_*i*_ = *exp*(*x*_*i*_* β*), *x*_*i*_ is a (*p* − 1) dimensional vector of covariates and *β* is a *p*-dimensional vector of coefficient of covariate or parameters, α is a parameter of GPR, *p* is a positive integer. Means and variance of GPR distribution are *E*(*Y*_*i*_|*x*_*i*_) = *µ*_*i*_ and Var(*Y*_*i*_|*x*_*i*_) = *µ*_*i*_ (1 + α*µ*_*i*_)^2^ respectively. If  α = 0 then GPR model reduce to the Poisson regression model.

Inference procedures through regression models are conducted by estimating parameters values based on available observational data. The parameters estimation methods for both SR and GPR models used maximum likelihood method. After obtaining the regression model results from the estimate parameters, the next step is to evaluate the regression model using the analysis of variance (ANOVA) table, which provides information about levels of variability within a regression model and form a basis for tests of significance. ANOVA calculations are shown in the analysis of variance table. The ANOVA table contains the *F* test statistic for testing the hypothesis that  *β* ≠  0 against the null hypothesis that *β* =  0. The *F* test is defined as the ratio of the mean square model and the means square error. If the ratio is large than there is evidence against the null hypothesis.

## Results

**Figure 2:**
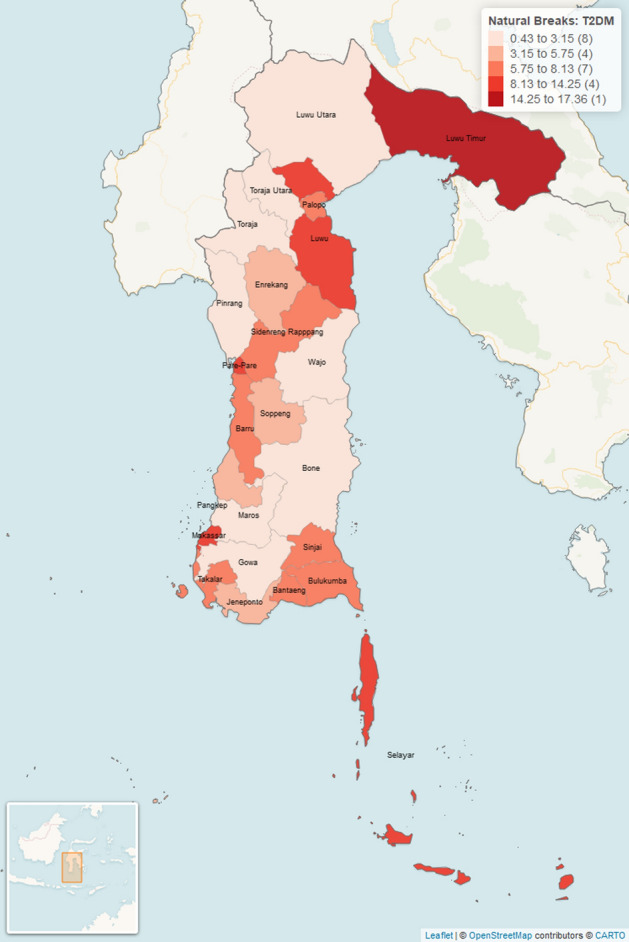
Prevalence rate of type 2 diabetes mellitus for the 2017–2018 period in South Sulawesi Province. The number in the parentheses indicated the number of districts/cities. This figure was generated by using R Basic version: 4.2.2 (https://cran.r-project.org/bin/windows/base/) and R Studio Desktop version: 2022.07.2 + 576 (https://posit.co/download/rstudio-desktop/) for windows operating system. We also utilized several packages to produce this figure such as sf package (https://r-spatial.github.io/sf/), dplyr package (https://github.com/tidyverse/dplyr), rgdal package (http://rgdal.r-forge.r-project.org/), tmap package (https://github.com/r-tmap/tmap) and leaflet package (https://rstudio.github.io/leaflet/). The details of district/city covered in this figure are listed as follows: (0.43 to 3.15): Luwu Utara, Toraja Utara, Toraja, Pinrang, Wajo, Bone, Maros, Gowa (8 districts/cities). (3.15 to 5.75): Enrekang, Soppeng, Pangkep, Jeneponto (4 districts/cities). (5.75 to 8.13): Palopo, Sidenreng Rappang, Barru, Takalar, Bantaeng, Bulukumba, Sinjai (7 districts/cities). (8.13 to 14.25): Luwu, Pare-Pare, Makassar, Selayar (4 districts/cities). (14.25 to 17.36): Luwu Timur (1 districts/cities).

The comparison of the prevalence rate values of T2DM in each district/city in the 2017–2018 period is shown in Table 1 and visualized in 5 groups as shown in Fig. 2 as follows:

**Table 1:** The prevalence rate of T2DM and hypertension (per 100,000 people) for the 2017 to 2018 period in South Sulawesi Province, Indonesia.

District/City	T2DM		Hypertension	
	N	Prevalence	N	Prevalence
Selayar	12	8.94	55	40.96
Bulukumba	32	7.65	163	38.96
Bantaeng	12	6.43	85	45.55
Jeneponto	16	4.42	81	22.39
Takalar	19	6.42	89	30.08
Gowa	21	2.76	106	13.94
Sinjai	19	7.83	80	32.97
Maros	9	2.57	98	28.01
Pangkep	18	5.41	119	35.77
Barru	13	7.49	84	48.38
Bone	21	2.78	144	19.08
Soppeng	8	3.53	104	45.86
Wajo	10	2.52	157	39.57
Sidenreng Rapppang	20	6.69	74	24.74
Pinrang	9	2.40	84	22.42
Enrekang	8	3.91	49	23.92
Luwu	34	9.47	181	50.39
Toraja	1	0.43	53	22.76
Luwu Utara	4	1.29	60	19.33
Luwu Timur	51	17.36	152	51.73
Toraja Utara	5	2.18	74	32.20
Makassar	127	8.42	396	26.26
Pare-Pare	16	11.13	50	34.79
Palopo	11	6.09	59	32.65
South Sulawesi Province (Total)	496	5.75	2597	32.61

Table 1 showed that the prevalence rate of T2DM for the 2017–2018 period was generally around 1–2 cases for every 100,000 population at a district/city in South-Sulawesi Province. The minimum, maximum, mean and standard deviation of prevalence rate of T2DM are 0.43, 17.36, 5.75 and 3.78 respectively (Table 2). The number of cases equal to three or more T2DM cases occured in Bulukumba and Sidenreng Rappang district (3–4 cases).

**Table 2:** Descriptive count and prevalence rate of T2DM and hypertension (per 100,000 people) for the 2017 to 2018 period in South Sulawesi Province, Indonesia.

Descriptive	T2DM		Hypertension	
	Count	Prevalence	Count	Prevalence
Mean	20.67	5.75	108.21	32.61
SD	25.12	3.78	72.36	10.77
Median	14.50	5.75	84.50	32.43
Minimal	1.00	0.43	49.00	13.94
Maximum	127.00	17.36	396.00	51.73

**Figure 3:**
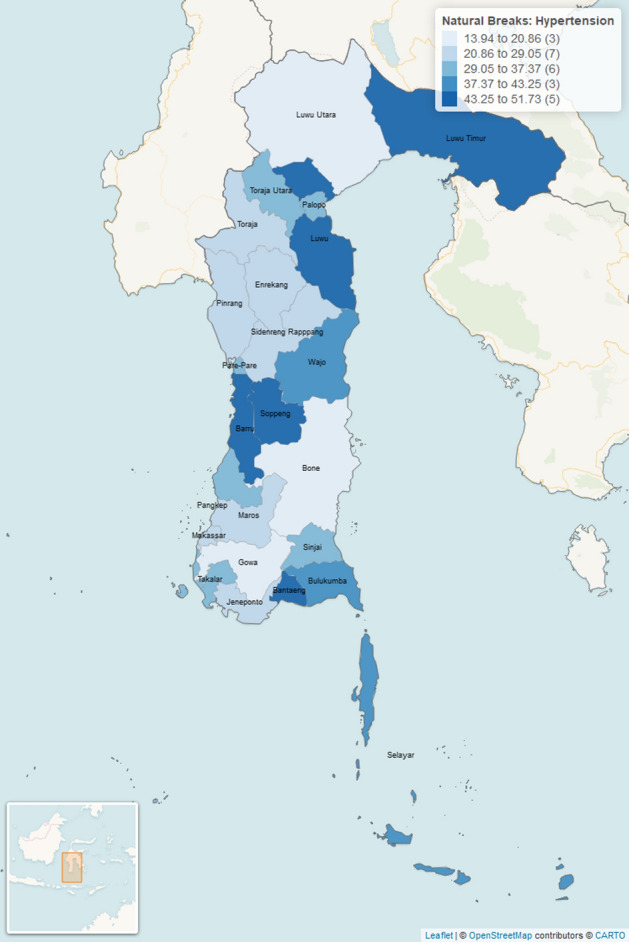
Prevalence rate of hypertension in the 2017–2018 period in South Sulawesi Province. The number in the parentheses indicated the number of districts/cities. This figure was generated by using R Basic version: 4.2.2 (https://cran.r-project.org/bin/windows/base/) and R Studio Desktop version: 2022.07.2 + 576 (https://posit.co/download/rstudio-desktop/) for windows operating system. We also utilized several packages to produce this figure such as sf package (https://r-spatial.github.io/sf/), dplyr package (https://github.com/tidyverse/dplyr), rgdal package (http://rgdal.r-forge.r-project.org/), tmap package (https://github.com/r-tmap/tmap) and leaflet package (https://rstudio.github.io/leaflet/). The details of city covered in this figure are listed as follows: (13.94 to 20.86): Luwu Utara, Gowa, Bone (3 districts/cities). (20.86 to 29.05): Toraja, Enrekang, Pinrang, Sidenreng Rappang, Maros, Makassar, Jeneponto (7 districts/cities). (29.05 to 37.37): Toraja Utara, Palopo, Pare-pare, Pangkep, Sinjai, Takalar (6 districts/cities). (37.37 to 43.25): Wajo, Bulukumba, Selayar (3 districts/cities). (43.25 to 51.73): Luwu Timur, Luwu, Soppeng, Barru, Bantaeng (5 districts/cities).

The prevalence rate of hypertension for the 2017–2018 period in each district/city is shown in Fig. 3 as follows:

In general, the prevalence rate of hypertension cases for the 2017–2018 period is 2–3 cases for every 100,000 population. Wajo, Luwu and Palopo city represents areas with a prevalence rate of 5 to 7 cases for every 100,000 population. The minimum, maximum, mean and standard deviation of prevalence rate of hypertension are 13.94, 51.73, 32.61 and 10.77, respectively (Table 2).

The results of the spatial cluster analysis using Global Moran's *I* to see the spatial cluster in the study area showed a score = − 0.0670 (*p* = 0.678) for the prevalence rate of T2DM and a score = − 0.0633 (*p* = 0.711) for the prevalence of hypertension. This indicates that the prevalence of T2DM and hypertension cases tends to occur randomly. The estimation parameter on the spatial cluster analysis is shown in Table 3.

**Table 3:** Spatial cluster analysis using global Moran's I test.

Variable	Global Moran's *I*	*p-*value	Conclusion
T2DM	− 0.0670	0.678	Not significant
Hypertension	− 0.0663	0.711	Not significant

Table 4 shows the results of the value of the estimated parameters in the Poisson regression model.

**Table 4:** The ANOVA of Poisson regression model.

Variable	Coefficient	SE	z-value	*p-*value	Conclusion
Intercept	5.22	0.14	36.82	< 0.001	Significant
T2DM	0.004	0.0005	7.56	< 0.001	Significant

Residual deviance: 1772.8 on 22 degrees of freedom.

The estimated value of the coefficient of T2DM was 0.005, the standard of error was 0.0005 and the *p-*value was < 0.001. However, the residual deviance was 1772.8 divided by 22 degrees of freedom earns more than one. It indicated an overdispersion of the models. For that matter, it was further used a GPR model approach to overcome the overdispersion problem above. The estimated parameter of the GPR is shown in the ANOVA Table 5.

**Table 5:** The ANOVA of generalized Poisson regression model.

Variable	Coefficient	SE	z-value	*p-*value	Conclusion
Intercept 1	2.16	0.17	36.82	< 0.001	Significant
Intercept 2	2.77	0.28	9.89	< 0.001	Significant
T2DM	0.005	0.0007	6.39	< 0.001	Significant

The GPR parameter estimation produced a standard error value of 0.0007 and a *p-*value of < 0.001, which indicated a significant impact between the number of T2DM on the number of hypertension.

## Discussion

While often have no obvious symptoms at first, T2DM and hypertension are two initial diseases on developing severe cardio- and cerebrovascular complications. Considering T2DM and hypertension are associated with single nucleotide polymorphisms (SNPs) genetic mutations^9^, the production of map to inform family or territorial clusters is important in effort to effectively detect T2DM and hypertension^10^.

By study conducted in Taiwan on 922 participants, 30 novel single nucleotide polymorphisms (SNPs) were associated with comorbid hypertension in T2DM patients adjusted for age and body mass index (*p-*value < 1 × 10^−4^). A cumulative genetic risk score consisting of 14 of the 38 SNPs is important for hypertension and increased propensity for systolic blood pressure and may contribute to hypertension in T2DM in this country^10^. Another study conducted in Malaysia involving 320 volunteers classified based on hypertension (163) and normotensive (157) conditions showed that TT genotype/T allele of the WNK4 gene resulted in a close relationship between hypertension and T2DM^11^. While genetic study remained limited in Indonesia, it is been documented that polymorphism of rs87148, especially CC genotype and C allele, and CAPN10 had a significant association with HbA1c level and increased T2DM vulnerability, respectively^12,13^.

In this study, we used sample data from patients who visited health care facilities which was registered by Social Security Administrator for Health (BPJS) in the 2017–2018 period. Our study showed that the prevalence of T2DM and hypertension in South Sulawesi Province was 1–2 and 2–3 patients for every 100,000 population, respectively. These values are slightly lower than the national average of 2% and 8.4% for T2DM and hypertension in the same period.

By using spatial analysis, we found that the distribution of T2DM and hypertension had same patterns. There was no correlation between one variable at one location and different variable at neighboring locations. The fact that both of the diseases are non-infectious disease may contribute to this result. Since the characteristics of the districts/cities in South Sulawesi Province are almost equal in terms of the human development index, community characteristics and health facilities, it is safe to propose that the distribution pattern of T2DM and hypertension is closely related to genetic and lifestyle. The observation of strong relationship on the gene-lifestyle interaction to develop T2DM and hypertension further corroborated the pivotal role of genetic and lifestyle factor on the risk of these diseases^14–16^.

Spatial regression analysis and classical regression found that the regression model of 70% and 71% could explain the variation of this finding. The results of the ANOVA table show that T2DM has a *p-*value of < 0.001. This indicates that T2DM has a significant linear relationship to increase hypertension. This is concurred with the research conducted by Akalu and Yitayeh (2020) on entire T2DM patients in the Ethiopian Debre Tabor Hospital that the prevalence of hypertension in T2DM patients was 59.5%^17^. Research conducted in Benghazi also showed that 85.6%, 54.2% and 56.3% prevalence of hypertension among DM patients^18^. In line with this finding, a study conducted by Tsimihodimos et al. in Mexico City for seven years showed that 16% to 46% of subjects were experiencing hypertensive; among participants the prevalence of T2DM is around 20–39%^19^. Moreover, half of the patients with T2DM also had hypertension in Japan^20^.

Several translational studies had explored the mechanism underlying the close relation between T2DM and hypertension. For instances, patients with T2DM have increased peripheral arterial resistance caused by vascular remodeling and increased body fluid volume associated with hyperinsulinemia and insulin resistance-induced hyperglycemia^17^. In addition, a recent study has identified GLP1R (glucagon-like peptide-1 receptor) expression in the carotid body (CBs) of spontaneously hypertensive rat as a novel signaling circuit that mediate hyperglycemia-induced peripheral chemoreflex sensitization, sympathetic overactivity and eventually exacerbate hypertensive condition^21^.

This study needs further research since the data was sourced from secondary data which released by BPJS. It is based on patient visitation and needs more description to primary characteristics, hence patients who do not participate in the national health insurance scheme may not be recorded. Although this study had involved all cities/districts in South Sulawesi Province, it is also important to note that this province only consisted of a small number (24) of cities/districts. In this regard, further study that utilize individual patient-level data aggregate to the grid cells (for instance 1 km × 1 km) instead of cities/districts may generate more specific high-risk areas and stronger and more reliable statistical analysis. Given that T2DM and hypertension are asymptomatic in the early stages, some people do not visit health care facilities. This phenomenon indeed would not be documented in health insurance records.

## Conclusions

A geospatial analysis of patients with T2DM and hypertension has been carried out in this study. There were two geospatial analyzes carried out, namely: spatial cluster analysis and spatial regression analysis. Globally, the number of T2DM and hypertension cases registered with *BPJS Kesehatan* tended to occur randomly. The results of the spatial regression analysis showed that the prevalence of T2DM can increase the number hypertension. This data may be used by policy makers to plan a comprehensive program to reduce the prevalence and risk of complications of these diseases.

## Data Availability

The datasets generated and/or analyzed during the current study are available from the corresponding author on reasonable request.
